# Digital therapeutics for depression in Germany: A systematic app review

**DOI:** 10.1016/j.invent.2026.100991

**Published:** 2026-08-25

**Authors:** A. Schmitz, S. Frey, E. Treis-Hoffmann, F. Hagendorf, B. Weltermann, A. Karimzadeh

**Affiliations:** a University of Bonn, University Hospital Bonn, Institute for Family Medicine, Bonn, Germany; b Private Practice Occupational Medicine, Organizational Psychology & Systemic Therapy, Munich and Starnberg, Germany

**Keywords:** Depression, DiGA, mHealth, iCBT, Guideline adherence, App quality, Digital therapeutics

## Abstract

**Background and objective:**

In Germany, digital health applications (DiGAs) approved for statutory health insurance are increasingly used in the treatment of depression, yet their quality, content, and adherence to evidence-based approaches and adherence to guidelines are poorly studied.

**Methods:**

DiGAs for depression were identified through a structured selection process and independently evaluated using a predefined assessment framework. Analyses included guideline conformity and the presence of internet-based cognitive behavioral therapy (iCBT) components. App quality was assessed using the Mobile Application Rating Scale (MARS), and functional characteristics were evaluated using the Institute for Healthcare Informatics functionality scoring. Interrater reliability was calculated using Fleiss' kappa.

**Results:**

Five DiGAs were included for the review. The overall degree of guideline conformity varied across applications (mean = 62.24%, SD = 26.74%, range = 22–72%), indicating marked differences in the implementation of guideline-recommended therapeutic content. Three DiGAs implemented all five iCBT components, whereas the remaining applications included only selected elements. Monitoring approaches ranged from comprehensive daily tracking to minimal symptom assessments without feedback. Individual therapeutic support via telemedicine varied from in-app coaching and crisis services to indirect integration via external healthcare professionals. Heterogeneity was also observed in structure and interactivity. App quality, as measured by the MARS, was moderate (mean = 3.8, SD = 0.4, range = 3.2–4.2), while functional design scores differed across applications (mean = 9, SD = 2, range = 7–11).

**Conclusion:**

DiGAs for depression differ substantially in their adherence to clinical guidelines, quality, and functional characteristics. These findings highlight the need for transparent and multidimensional evaluations to support informed clinical decision-making.

## Highlights

• Five DiGAs for depression were systematically evaluated.
• Degree of guideline conformity varied across applications.
• Coverage of core iCBT components differed between DiGAs.
• App quality was moderate, with high functionality ratings.
• A multidimensional framework was applied for DiGA evaluation.

## 1 Introduction

Depressive disorders are among the most prevalent mental health conditions worldwide and represent a leading cause of disability, affecting more than 300 million people globally (Institute for Health Metrics and Evaluation, 2021; Cuijpers et al., 2023). In Germany, depression poses a substantial public health burden, with administrative data indicating a diagnosed prevalence of 17% among adults receiving outpatient care and annual direct health care costs of approximately €9.5 billion. (Robert Koch Institut, 2024; Wissenschaftliches Institut der AOK, 2024)

Evidence-based treatment of depression typically involves pharmacological and psychotherapeutic approaches, often applied in combination. Clinical practice guidelines recommend a stepped-care model depending on symptom severity, including antidepressant medication, psychotherapy (e.g. cognitive behavioral therapy CBT) and psychoeducational interventions (Bundesärztekammer (BÄK), 2022). While these treatments have demonstrated efficacy, barriers to access remain substantial (Cuijpers et al., 2023). Limited availability of therapy, long waiting times, stigma and geographical disparities contribute to gaps in care provision (Bundespsychotherapeutenkammer, 2021).

In response to these challenges, digital health interventions have gained increasing attention as scalable and accessible treatment options. Internet- and mobile-based interventions have demonstrated effectiveness in reducing symptoms (Karyotaki et al., 2021). Such interventions are now incorporated into clinical guidelines as low-threshold treatment options that can complement traditional care (Bundesärztekammer (BÄK), 2022).

Germany has established a unique regulatory framework, the fast-track process, for Digital Health Applications (DiGA). Introduced through the Digital Healthcare Act, DiGAs can be prescribed by physicians and reimbursed by statutory health insurance following evaluation by the Federal Institute for Drugs and Medical Devices (BfArM) (Federal Office of Justice, 2024). Applications may initially be granted a provisional listing if they fulfil the required criteria for safety, functionality, quality, data protection, data security, and interoperability, while evidence on positive health care effects is still being generated. During this period, manufacturers are required to conduct comparative clinical studies to demonstrate positive health care effects. Permanent listing requires sufficient evidence of these effects, typically demonstrated in clinical trials, after which reimbursement is maintained (Federal Institute for Drugs and Medical Devices, 2025). While the DiGA fast-track has been recognized as an innovative regulatory approach to accelerate patient access to evidence-based digital interventions, it has also been subject to ongoing discussion regarding the strength of the required evidence at the time of provisional listing, pricing during evaluation period, and the long-term sustainability of the reimbursement model. (Schmidt et al., 2024)

Within routine care, the uptake of DiGAs has expanded considerably since their introduction. By the end of 2025, approximately 1.6 million DiGA activation codes had been redeemed in Germany, including around 695,000 redeemed activation codes in 2025 alone, indicating a substantial increase in routine use (GKV-Spitzenverband, 2026). Mental health applications accounted for approximately 426,000 redeemed activation codes in 2025 (27% of all utilizations), with deprexis ranking among the most frequently used DiGAs overall (6.1% of all redeemed activation codes) (GKV-Spitzenverband, 2026). Health insurance data further indicate that DiGAs are often integrated early into the care pathway, with approximately 29.5% of individuals with mental and behavioral disorders receiving their first DiGA-relevant diagnosis within the three months preceding initial DiGA use (Techniker Krankenkasse, 2024).

A substantial body of evidence on digital interventions for depression has emerged, including randomized controlled trials and meta-analyses demonstrating small to moderate effects (Klein et al., 2016; Krämer et al., 2022; Miegel et al., 2019; Haaf et al., 2024). In parallel, studies have evaluated the quality of commercially available mental health applications using standardized instruments such as the Mobile Application Rating Scale (MARS) as well as an initiative such as the Mobile Health App Database (MHAD), which provides expert-rated quality assessments of mHealth applications to support informed decision making (Universität Ulm, 2023; Kaveladze et al., 2022; Myers et al., 2020; Terhorst et al., 2018). However, these evaluations have primarily focused on apps available in commercial app stores rather than regulated digital therapeutics. Existing literature also highlights recurring methodological concerns, including limitations of the control conditions. Many trials compare the intervention with waiting-list or usual-care conditions, making it difficult to distinguish intervention-specific effects from the effects of attention, treatment expectations or access to an additional care option (Cuijpers et al., 2024). Further concerns include high attrition rates, lack of blinding and reliance on patient-reported outcomes (Kolominsky-Rabas et al., 2022; Schreiter et al., 2023; Sippli et al., 2025).

Moreover, prior research has primarily focused on clinical efficacy and regulatory approval studies, while other relevant dimensions such as therapeutic content, adherence to clinical guidelines, functional characteristics and overall app quality have not been investigated (Sippli et al., 2025). It remains unclear to what extent these applications implement evidence-based therapeutic strategies and whether their functional and qualitative characteristics support treatment delivery. Moreover, there is currently no standardized or comprehensive framework for the systematic evaluation of DiGAs that integrates multiple relevant dimensions, such as guideline adherence, therapeutic content, app quality, and functional characteristics. Existing evaluation approaches typically focus on isolated aspects, for example clinical efficacy or usability, and therefore do not allow for a comprehensive assessment or valid comparison across applications. In light of these considerations, there is a need for a structured and multidimensional approach that captures the complexity of digital interventions for depression. The absence of such a unified framework has motivated the development of the present evaluation approach, which combines established assessment tools with guideline-based criteria to enable a more comprehensive analysis of DiGAs.

The present study aims to provide a comprehensive evaluation of DiGAs for depression by addressing the research question: To what extent do currently available DiGAs for depression adhere to clinical treatment guidelines and how do they perform in terms of quality and functional characteristics? Beyond the German context, this study illustrates how regulated digital therapeutics can be evaluated using a multidimensional framework that integrates therapeutic content, guideline adherence, app quality and functional characteristics, thereby providing insights relevant to emerging regulatory frameworks for digital therapeutics internationally.

## 2 Methods

The methodological approach of this systematic application review was pre-registered in PROSPERO (Schmitz et al., 2024). The registered protocol included applications targeting both depressive disorders and generalized anxiety disorder (GAD) (Schmitz et al., 2024; Schmitz et al., 2025). During the review process, it became apparent that only a very limited number of permanently listed DiGAs were available for GAD, precluding a meaningful multidimensional comparison across applications. To ensure a focused and sufficiently comparable evaluation, the present study was therefore restricted to applications targeting depressive disorders. This deviation from the registered protocol was made before data synthesizes. No further deviations from the registered protocol occurred. (Schmitz et al., 2025) The review design was guided by the structured approach proposed by Gasteiger et al. 2023 (Gasteiger et al., 2023).

The interdisciplinary research team consisted of five reviewers with expertise in digital health and mental health research. Application testing and data extraction were independently performed by three reviewers (SF, ETH and AS). Any disagreements were resolved through consensus discussion involving additional reviewers (AK or FH).

### 2.1 Search strategy and eligibility criteria

To identify eligible applications for the review, the DiGA directory was used as the sole source, as it provides a comprehensive listing of all approved DiGAs (Federal Institute for Drugs and Medical Devices, 2026). Following the approach described by Gasteiger et al. (2023) the official DiGA directory was first filtered for ‘mental health’ and ‘permanently listed’ applications (Gasteiger et al., 2023). Subsequently, titles, indications and manufacturer-provided information were screened. No search string was required due to the structured nature of the directory. The remaining applications were then downloaded, and final eligibility was confirmed through a full app assessment. Duplication removal was not necessary, as only a single source was used. Eligibility criteria were predefined in the registered protocol according to the Target user groups, Evaluation focus, Connectedness, Health Domain (TECH) framework:

Target group: adults (> 18 years) with symptoms of depressive disorders

– Evaluation focus: psychotherapeutic interventions
– Connectedness: standalone applications, optionally connected to other technologies but primarily functioning independently
– Health domain: at least one indication according to the International Classification of Diseases codes: F32.0; F32.1; F32.2; F33.0; F33.1; F33.2; F34.1

Interrater agreement was quantified using Cohen's kappa for two-reviewer procedures. (Cohen, 1960) The initial search was conducted in July 2024. The search was repeated in July 2025 using the identical search strategy and eligibility criteria.

### 2.2 Evaluation procedure

To standardize the evaluation process, reviewers followed predefined patient vignettes (Schmitz et al., 2025). These vignettes were used to enhance consistency and objectivity during application usage. The vignettes were derived from published literature and represented three cases with varying symptom severity and clinical courses (Andrews, 2009; Miller et al., 1997; Persons et al., 2001). This approach ensured that applications were assessed across a range of relevant user scenarios. Each reviewer independently completed all modules and exercises available within every application using full-access accounts provided by the manufacturers before completing the assessments.

Applications were tested using iPhone 15 Pro, iPhone 12 and iPhone SE devices (iOS versions 26.2 and subsequent minor versions). Web-based applications were accessed via a Windows 11 Education environment (version 23H2) using Microsoft Edge and Mac OS Tahoe using Safari 26.5. The data extraction sheet and assessment procedures were pilot tested prior to the review.

### 2.3 Data generation

General application characteristics were independently extracted by two reviewers using a predefined data extraction sheet. The extraction framework was adapted from Arnhold et al. (2014) and modified to reflect the specific context of regulated digital therapeutics (Arnhold et al., 2014). Extracted variables included general app information, operating systems, developer characteristics, pricing structures, target populations, interoperability, and data security features.

To systematically evaluate therapeutic content, a structured guideline conformity checklist (GLCC) was developed for this study. The GLCC was derived from the German national clinical practice guideline for depressive disorders (S3-guideline) and operationalized into measurable items reflecting evidence-based therapeutic strategies (Bundesärztekammer (BÄK), 2022). The checklist aimed to capture the extent to which core guideline-recommended components were implemented within the applications, including psychoeducation, behavioral activation, cognitive restructuring, and relapse prevention (Schmitz et al., 2025). In addition to the structured ratings, reviewers documented narrative observations in a free-text field (“additional synthesis”). These narrative summaries were synthesized into the qualitative application description and subsequently reviewed and agreed upon by all reviewers. Where relevant, additional observations that emerged during consensus meetings were incorporated to ensure that descriptions accurately reflected the reviewers shared assessment of each application.

In parallel, a second checklist was constructed to assess the presence of key elements of internet-based cognitive behavioral therapy (iCBT), based on established literature (Furukawa et al., 2018; Furukawa et al., 2021; Watkins et al., 2023). This instrument included components such as modular structure, homework assignments, symptom monitoring, and feedback mechanisms.

Both checklists were independently applied by three reviewers. Items were rated using a three-level coding scheme (0 = absent, 1 = present, tbd = unclear). Interrater reliability was quantified using Fleiss' kappa (Fleiss, 1971). Discrepancies were resolved through consensus discussions with a fourth reviewer.

Application functionalities were assessed using the framework developed by the IMS Institute for Healthcare Informatics (Institute for Healthcare Informatics, 2023). This standardized instrument comprises 11 predefined functional categories (e.g., inform, instruct, record, guide), allowing for a structured evaluation of app capabilities. Each function was coded dichotomously (present vs. absent). Interrater agreement was again assessed using Fleiss' kappa to ensure reliability of ratings.

Application quality was evaluated using MARS, a widely used and validated multidimensional instrument for assessing mobile health applications (Terhorst et al., 2020). Before commencing the assessments, all reviewers completed an online MARS training to ensure standardized application of the rating assessment. MARS captures four objective quality domains (engagement, functionality, aesthetics, and information quality) as well as a subjective quality rating. All reviewers completed the assessment independently via a standardized online survey platform (unipark).

Data analysis was descriptive. Quantitative summaries were calculated using Microsoft Excel, including means, standard deviations, and ranges for continuous variables and frequencies and percentages for categorical variables. Interrater reliability statistics (Fleiss' kappa) were computed using the statistical software R (Posit team, 2025).

## 3 Results

**Fig. 1:**
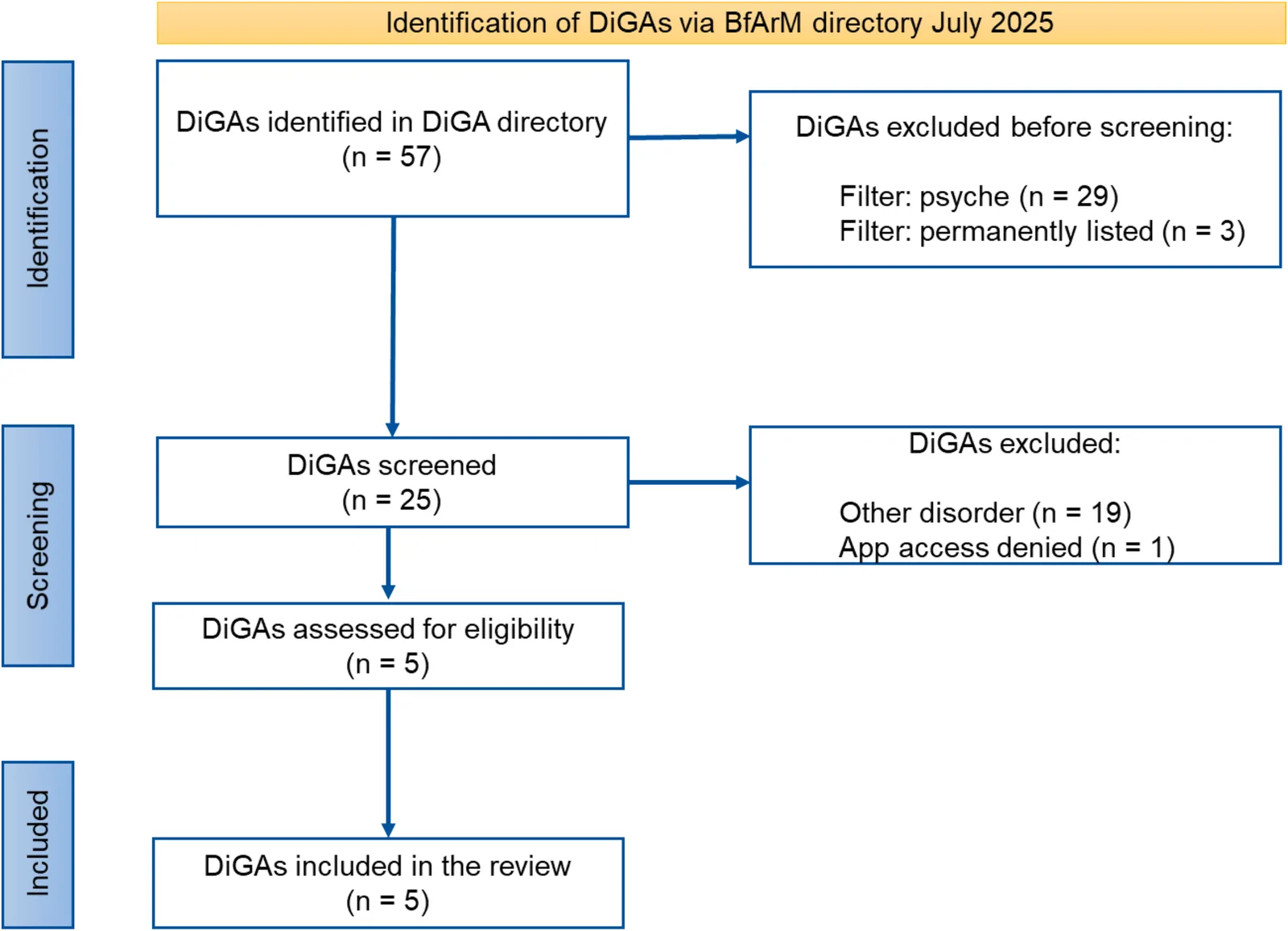
DiGA identification and selection process – flow diagram.

As of July 2025 57 DiGAs were listed in the DiGA directory. Of these, 25 DiGAs were permanently listed for psychiatric disorders (Fig. 1). During the screening process, 19 DiGAs were excluded due to targeting other mental disorders. Five DiGAs met the eligibility criteria and were included in the review. One application (elona therapy Depression) passed the screening, but could not be evaluated, because the manufacturer did not provide access to the application despite repeated requests. The two reviewers agreed fully (Cohen's kappa = 1). The flow diagram reflects the updated search conducted in July 2025. Compared with the initial search in July 2024, two additional permanently listed DiGAs (My7steps App and elona therapy Depression) were identified and included in the screening process.

Included DiGAs targeting depression were first listed in the DiGA directory between 2020 and 2023 (Table 1). Costs ranged from 199.00 to 228.00 EUR for a 3-month usage period (mean = 215.20 EUR, SD = 12.14). All applications were available as web-based desktop applications (100%), with a subset additionally offered as mobile applications (40%). The number of indications and contraindications varied substantial across applications. However, all DiGAs targeted unipolar depressive disorders of mild to moderate severity. Two applications also included patients with dysthymia. While all DiGAs were intended for adults aged 18 to 65 years, two DiGAs extend their targeted patient group to individuals over 65 years. The number of contraindications varied considerably, ranging from 5 to 33 contraindications. Three DiGAs reported five contraindications, whereas two applications listed higher numbers (29 and 33 contraindications), indicating two distinct value clusters. Detailed information on indications, contraindications and further app characteristics is provided in Supplementary Material S1.

DiGAs were all used in German. Additional language options ranged from 1 to 12. One DiGA offered only German, two DiGAs offered German and English, whereas two applications provided substantially more language options (9 and 12). Table 2 summarizes the full distribution of available languages across the DiGAs.

**Table 1:** General application characteristics of the selected DiGAs for depression (*N* = 5).

Category	*Subcategory*	*n*	*%*
Number of languages	Only German	1	20
	German and English	2	40
	German, English and others	2	40
Year of listing	2020	1	20
	2021	2	40
	2022	1	20
	2023	1	20
No. of indications	4	3	60
	6	1	20
	7	1	20
No. of contraindications	5	3	60
	29	1	20
	33	1	20
Application form	Web application	5	100
	Mobile application (iOS and Android)	2	40
	Both	2	40
Age	18–65 years	5	100
	> 65 years	2	40
Gender	Female, male, non-binary	5	100
Risk classification (MDR)	I	5	100
Costs	< 200€	1	20
	200–300€	4	80

*Abbreviations*. N–Number of DiGAs; %–Percentage; MDR – Medical Device Regulation

**Table 2:** Language availability of selected DiGAs for depression (*N* = 5).

	deprexis	Novego: Depressionen bewältigen	My7steps App	edupression.com	Selfapys Online-Kurs bei Depression
German	✓	✓	✓	✓	✓
English	✓		✓	✓	✓
French	✓		✓		
Greek	✓		✓		
Italian	✓		✓		
Portuguese	✓		✓		
Spanish	✓		✓		
Arabic			✓		
Chinese	✓				
Persian			✓		
Russian			✓		
Swedish	✓				
Turkish			✓		
Ukrainian			✓		

### 3.1 Functional characteristics of the DiGAs

**Fig. 2:**
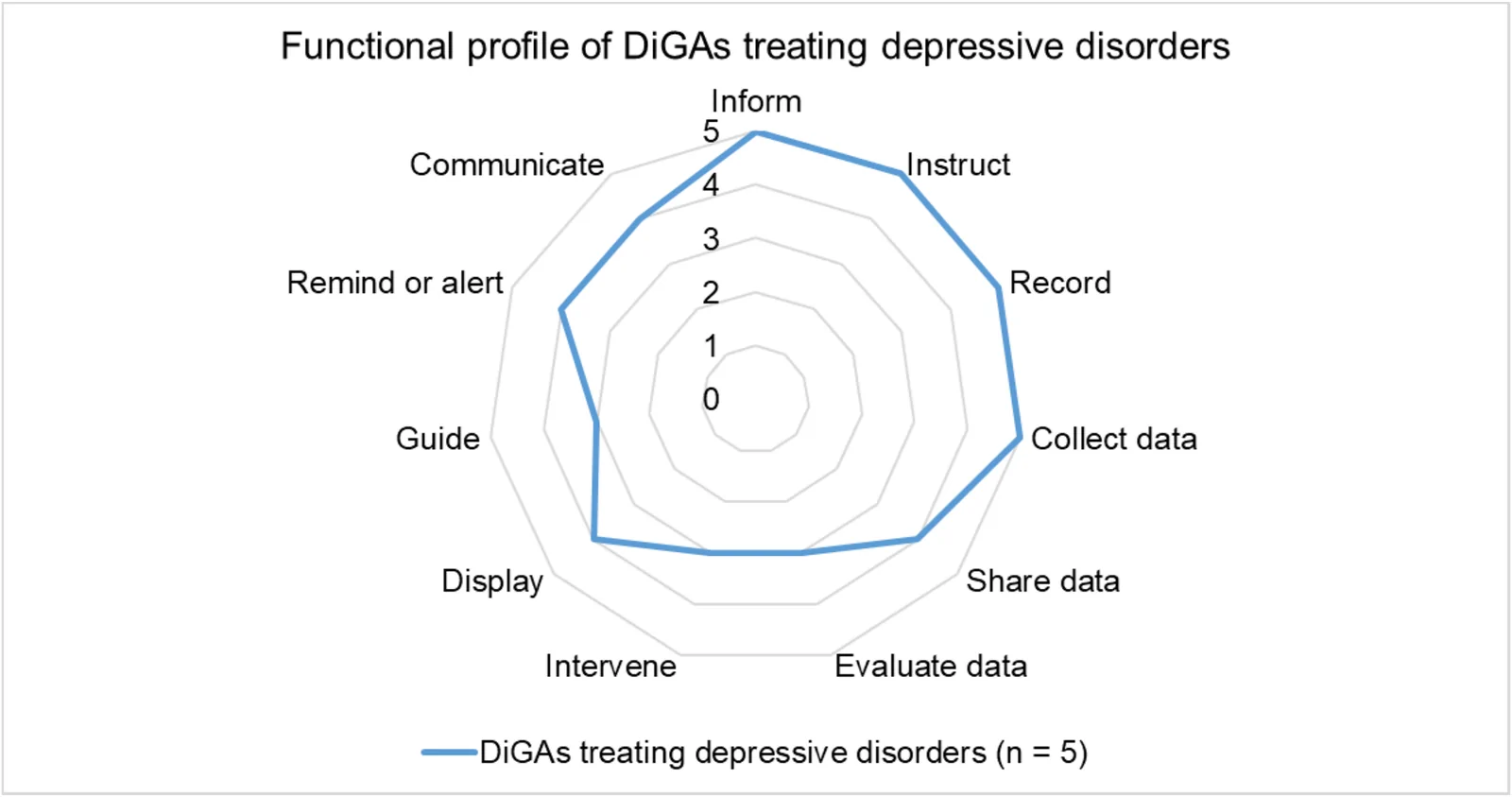
Aggerated functional profile of DiGAs treating depressive disorder (*N* = 5). Values indicate the number of DiGAs (0–5) implementing each functionality.

Three reviewers independently explored each included DiGA during active use to determine the range of implemented functions. Functional classification was conducted using the IMS Institute for Healthcare Informatics framework (Institute for Healthcare Informatics, 2023). Interrater reliability indicated moderate agreement (Fleiss' kappa = 0.48, *p* < 0.05). On average, applications provided nine functions (SD = 2, range = 7–11). All applications consistently included the functions inform, instruct, record and collect data. Fig. 2 provides an aggregated overview of the functional characteristics across all included DiGAs. The most functionally comprehensive applications were My7steps App and edupression.com, whereas deprexis and Selfapys Online-Kurs bei Depression provided the lowest functional scope. Detailed information on the functional characteristics of the included DiGAs is provided in the Supplementary Material S4.

### 3.2 Degree of guideline conformity

**Fig. 3:**
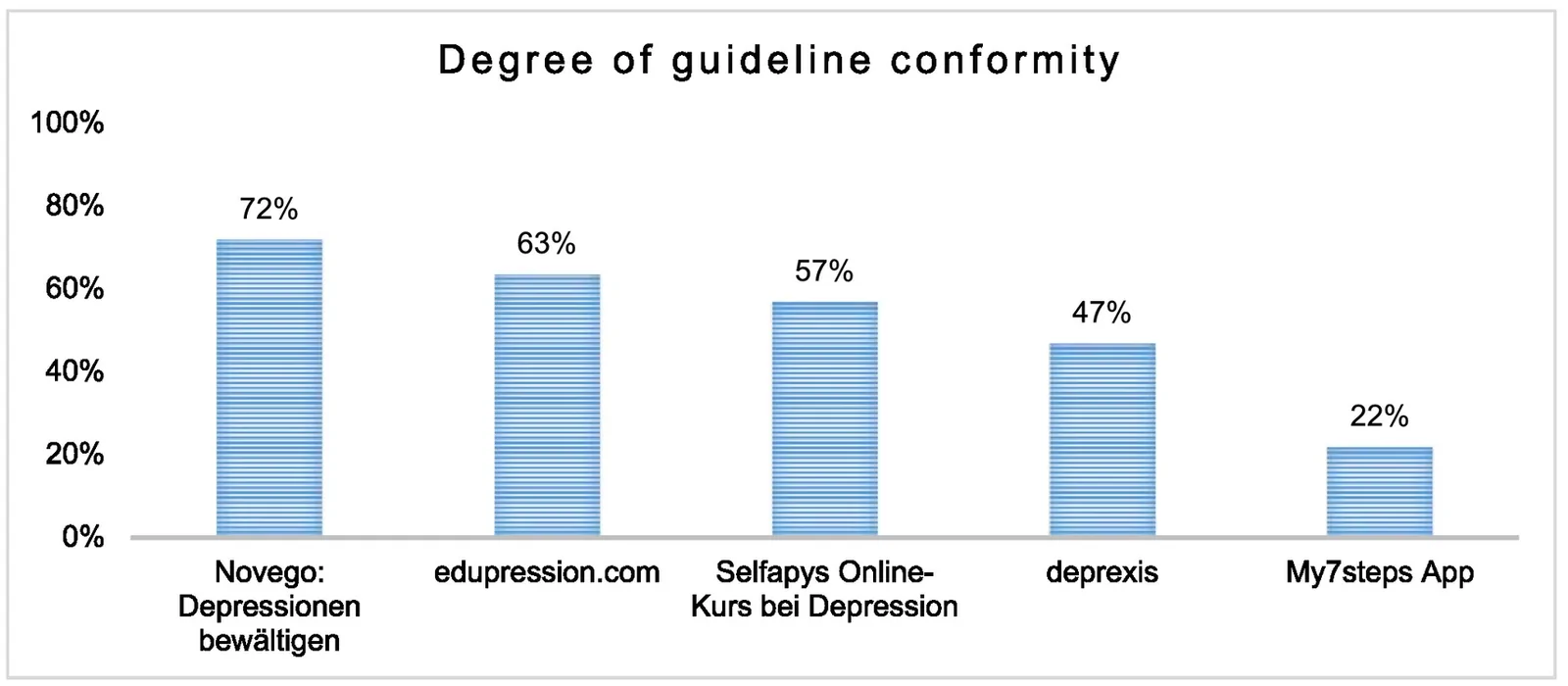
Degree of guideline conformity (%) for each DiGA (*N* = 5).

The assessment of guideline conformity revealed differences among the DiGAs examined (Fig. 3). In terms of overall score, Novego: Depressionen bewältigen achieved the highest level of compliance at 72%, followed by edupression.com (63%) and the Selfapys Online-Kurs bei Depression (57%), deprexis scored 47%, placing it in the middle range, while the My7steps App showed lower conformity at 22%. Across all applications, the average degree of guideline conformity was 62%. Reviewers agreed substantially (Fleiss' kappa = 0.75, *p* < 0.001). The completed consensus GLCC is displayed in the Supplementary Material S2.

The analysis of categories revealed also differences in the DiGA contents (Table 3). On average, just under half (48%) of the items in the category psychoeducation and training were implemented, with edupression.com showing the highest coverage (80%) and the My7steps App the lowest (13%). Elements of cognitive behavioral therapy were strongly represented overall (mean 80%), with full implementation in deprexis and Novego: Depressionen bewältigen, while My7steps App achieved significantly lower rates here (40%). Psychoanalytically based methods played a minor role overall (20%) and were completely absent from several applications.

**Table 3:** Degree of guideline conformity per category and DiGA (*N* = 5).

	deprexis	Novego: Depressionen bewältigen	My7steps App	edupression.com	Selfapys Online-Kurs bei Depression	Mean Overall
	*%*	*%*	*%*	*%*	*%*	*%*
Psychoeducation and training (*n* = 30)	37	63	13	80	47	48
Cognitive behavioral therapy (n = 5)	100	100	40	80	80	80
Psychoanalytically based procedures (n = 4)	0	50	0	25	25	20
Systemic therapies (n = 4)	25	75	25	0	75	40
Cross-procedural impact factors (*n* = 10)	50	80	40	30	50	50
Monitoring (*n* = 1)	100	100	100	100	100	100
Therapeutic support (n = 1)	0	100	100	100	100	80
Low-intensity interventions (n = 5)	100	100	20	80	100	80
Overall	47	72	22	63	57	62

*Abbreviations.* n = number of items in GLCC category, %–percentage.

Systemic therapeutic approaches were incorporated in 40% of the applications on average, with particularly high implementation rates in Novego: Depressionen bewältigen and the Selfapy Online-Kurs bei Depression (75% each). Cross-sectional factors were found in about half of the applications (50%). The included DiGAs used diverse monitoring strategies to support adherence and assess treatment outcomes. While all apps featured at least one monitoring component, their scope varied considerably. The DiGA edupression.com provided the most comprehensive approach, with daily tracking of symptoms, treatment progress, lifestyle factors, and detailed medication data. In contrast, Novego: Depressionen bewältigen collected symptom data without offering user feedback and My7steps App displayed a change in self-rated symptom severity over time alongside optimism measures. Some DiGAs also supplemented monitoring with downloadable materials such as logs or diaries to support daily use.

Therapeutic support was also widespread (80%), with the exception of deprexis, where therapeutic support was not present. My7steps App provided coaching with a focus on psychosocial guidance during the intervention, whereas Novego: Depressionen bewältigen emphasized crisis support through a 24/7 hotline access for acute situations. The DiGA edupression.com did not offer direct in-app support but instead offered to share user data with treating professionals, who could review symptom trends and therapy progress.

Low-threshold interventions were frequently integrated overall (80%), with deprexis, Novego: Depressionen bewältigen and Selfapys Online-Kurs bei Depression showing full conformity. In contrast, the My7steps App showed lower implementation in this area as well (20%).

In summary, the results indicate a generally good alignment with guideline-based content, though this varies greatly among the applications, with clear strength in the areas of cognitive behavioral therapy and monitoring.

As part of the guideline conformity assessment, reviewers also added qualitative observations:

• deprexis uses a structured, dialogue-style format with predefined answer options, limiting input to fixed responses. It includes additional content (e.g., on dreams), and some scenarios appear more tailored to younger users.
• Novego: Depressionen bewältigen combines psychoeducation with behavioral activation across twelve weekly modules using text, audio, and video. It strongly promotes activity increase, recommends consulting a physician, and suggests follow-up psychotherapy. Supplementary materials and additional topics (e.g., light therapy, sensitive issues) are included.
• My7steps App follows a fixed seven-step structure requiring continuous engagement, including reflections, structured inputs, and behavioral tasks, with prompts to contact in-app therapists at set points.
• Selfapys Online-Kurs bei Depression is mainly text-based with some videos and limited interactivity. Although free-text input is possible, content progression remains fixed. Most materials are downloadable as PDFs.
• edupression.com focuses heavily on psychoeducation, offering extensive video content and in-depth explanations (e.g., pharmacology). It emphasizes self-directed use, with minimal interactive guidance and additional downloadable summaries.

### 3.3 Adherence to iCBT components

Reviewers assessed the presence of iCBT components across DiGAs (Table 4). Interrater reliability was fair (Fleiss' kappa = 0.31, *p* = 0.008), though interpretation is limited given the small number of rated items (*n* = 25) and the dichotomous rating format. Full coverage of all iCBT elements was identified in deprexis, Novego: Depressionen bewältigen, and Selfapys Online-Kurs bei Depression. In contrast, My7steps App included only psychoeducation and behavioral activation. Although edupression.com covered a similar number of behavioral therapy components as Selfapys Online-Kurs bei Depression in the guideline-based assessment, it incorporated fewer iCBT elements overall (3 vs. 5).

**Table 4:** Implementation of iCBT components of included DiGAs (*N* = 5).

	deprexis	Novego: Depressionen bewältigen	My7steps App	edupression.com	Selfapys Online-Kurs bei Depression
Psychoeducation about depression	+	+	+	+	+
Cognitive restructuring	+	+	−	+	+
Behavioral activation	+	+	+	+	+
Interpersonal skill training	+	+	−	−	+
Problem solving	+	+	−	−	+

Note. + available; − not available

**Table 5:** Quality ratings using MARS for selected DiGAs treating depression (*N* = 5).

	Engagement	Functionality	Aesthetics	Information	App Quality	App Subjective Quality
	*M (Range)*	*M (Range)*	*M (Range)*	*M (Range)*	*M (Range)*	*M (Range)*
deprexis	3.1 (2.8–3.6)	4.8 (4.5–5)	4.3 (3.6–4.6)	4.5 (4–4.8)	4.2 (3.8–4.5)	3.0 (2.7–3.2)
Novego: Depressionen bewältigen	3.5 (3.4–3.6)	4.6 (4–5)	4.0 (3.6–4.6)	4.2 (4.1–4.3)	4.1 (3.8–4.1)	2.7 (1.7–3.5)
My7steps App	3.3 (2.6–3.8)	4.3 (3–5)	3.8 (3.3–4)	3.7 (3.4–3.9)	3.8 (3.7–3.9)	2.3 (1.7–2.5)
Selfapys Online-Kurs bei Depression	3.4 (3–3.8)	3.9 (3.8–4)	4.1 (4–4.3)	4.2 (3.9–4.4)	3.9 (3.8–4.1)	2.9 (2.8–3.3)
edupression.com	2.6 (2–3.3)	3.6 (2.5–4.5)	3.44 (2.5–4.5)	3.33 (3.3–3.4)	3.2 (2.8–3.9)	1.9 (3.3–3.4)

*Abbreviations. M* – Mean.

### 3.4 Expert assessment of app quality

The assessment of app quality based on the individual MARS dimension identified a high variety across permanently listed DiGAs (Table 5). For functionality, the mean scores fell within a range between 3.6 and 4.8. The highest functional score was observed for deprexis, while edupression.com showed the lowest level of function. However, all DiGAs reached good scores. The overall app quality score ranged from 3.2 to 4.2. The highest overall ratings were given to deprexis (mea*n* = 4.2) and Novego: Depressionen bewältigen (mean = 4.1), while edupression.com had the lowest overall quality with a mean of 3.2. Subjective quality was consistently lower compared to the other dimensions. The mean scores ranged from 1.9 to 3.0. The DiGAs deprexis (mean = 3.0) and Selfapys Online-Kurs bei Depression achieved the highest scores, while edupression.com (mean = 1.9) received the lowest rating. Overall, subjective user satisfaction was limited despite predominantly good functional and content quality. The complete MARS assessment is displayed in the Supplementary Material S3.

## 4 Discussion

This study evaluated five DiGAs for depressive disorders listed in the German DiGA directory and identified variability across applications regarding therapeutic content, degree of guideline conformity, functionality and app quality. While all included applications addressed depressive symptoms using cognitive behavioral principles to some extent, considerable differences emerged in the depth of therapeutic implementation and overall structure of the interventions. Guideline conformity showed variation between applications (mean = 62%, SD = 26%, range = 22–72%), indicating inconsistent implementation of evidence-based treatment recommendations.

App quality, assessed with the MARS, was overall moderate (mean = 3.8, SD = 0.4), with high scores in functionality but more variable ratings regarding engagement and subjective quality. The DiGAs differed in the number of functions (mean = 9, SD = 2), interactivity, monitoring intensity and therapeutic support. Some applications provided comprehensive, structured interventions with ongoing monitoring and interactive exercises, while other DiGAs focused on psychoeducational or self-guided approaches with lower levels of in-app guidance.

Our findings extend previous MARS-based evaluations of depression applications. Before the introduction of the DiGA pathway, Terhorst et al. (2018) evaluated German-language depression apps available in commercial app stores and reported a mean overall MARS score of 3.01 (SD = 0.56) (Terhorst et al., 2018). More recent evaluations of commercially available depression applications reported a mean MARS score of 3.5 (Myers et al., 2020), while broader evaluations of mental health applications yielded a comparable mean score of 3.85 (Kaveladze et al., 2022). In comparison, the permanently listed DiGAs included in the present review achieved a mean MARS score of 3.8. Overall, the observed MARS scores were within the range reported for mental health applications in previous MARS-based evaluations and slightly higher than those reported for commercially available depression applications.

These findings not only reflect the heterogeneity of digital mental health interventions within the German DiGA landscape but also provide insights into the strengths and limitations of regulatory approval pathways for digital therapeutics. The variability between the officially approved DiGAs for depression is evident, despite their inclusion within the same regulatory framework. The German DiGA fast-track established transparent minimum requirements regarding data protection, safety, interoperability, quality and evidence of positive health care effects (Federal Institute for Drugs and Medical Devices, 2025). However, the present findings demonstrate that applications fulfilling these regulatory requirements may still differ substantially. Consequently, regulatory approval should be interpreted as conformation that minimum regulatory and evidentiary standards have been met rather than as an indication that therapeutically comparable interventions are available within the same indication. As a result, applications targeting the same indication may differ in therapeutic complexity and intended use.

Beyond the German healthcare system, these findings are relevant for other countries. Several European health care systems, including England (NHS Early Value Assessment), Belgium (mHealthBelgium), and France (PECAN) have introduced structured evaluation pathways for digital therapeutics (Arcà et al., 2025; Tokarska et al., 2023). Our findings demonstrate that regulatory approval and reimbursement should be complemented by multidimensional evaluations addressing therapeutic content, guideline adherence, functionality and app quality as these aspects are not necessarily captured by regulatory requirements alone. Further such an approach may support future comparative evaluations of regulated digital therapeutics across different healthcare systems.

The functional and content related differences may have practical implications for clinical implementation and prescription decisions. Since mobile health applications are recommended in the national care guideline for depressive disorders, physicians and psychotherapists are required to select appropriate applications for individual patients despite limited standardized information on the application's characteristics (Bundesärztekammer (BÄK), 2022). The variability identified complicates comparability between DiGAs and challenges evidence-informed prescription for routine care. Available information primarily focuses on regulatory approval and evidence of effectiveness rather than detailed therapeutic characteristics (Federal Institute for Drugs and Medical Devices, 2026). This may be specifically relevant for patients with differing levels of types and severity of symptoms, digital literacy, motivation and/or need for therapeutic support. Thus, prescribing a DiGA should extend beyond the approved indication and additionally consider therapeutic characteristics.

Furthermore, differences in intended usage duration and intervention depth may influence both clinical usefulness and user adherence. More comprehensive applications may provide broader therapeutic benefits through structured behavioral exercises, symptom tracking and repeated reflection tasks, whereas shorter less interactive interventions may reduce user burden and improve accessibility for some individuals. Greater therapeutic complexity may place greater cognitive and motivational demands which could represent a barrier for individuals experiencing core depressive symptoms such as impaired motivation, fatigue or loss of energy.

The evaluated DiGAs also differed regarding language availability, which may have implications for equitable access to digital mental health care. While some applications provided multilingual content, others were available exclusively in German. Such differences may limit accessibility for individuals with limited German proficiency and contribute to disparities in the utilization of DiGAs across population groups.

### 4.1 Strength and limitations

The analysis applied a structured and multidimensional evaluation framework combining assessments of guideline conformity, implementation of iCBT components, app quality, and functional characteristics. This approach enabled a comprehensive evaluation of DiGAs beyond effectiveness alone and allowed the comparison of therapeutic, technical and quality aspects across applications.

The inclusion of multiple evaluation dimensions provides a more differentiated understanding of the current DiGA landscape for depressive disorders. Previous evaluations of digital mental health interventions have focused primarily on effectiveness or usability (Haaf et al., 2024; Kolominsky-Rabas et al., 2022; Schreiter et al., 2023; Sippli et al., 2025). The present study additionally considered the implementation of evidence-based therapeutic components, monitoring approaches, and functional design characteristics. This multidimensional perspective supports a comprehensive interpretation of differences between applications and their potential implications for routine care.

When interpreting the findings some limitations should be considered. The number of included DiGAs was limited, reflecting the currently available applications for depressive disorders listed in the DiGA directory. Consequently, the findings may not be generalizable to other digital mental health interventions or future DiGAs. In addition, one eligible DiGA could not be included because access was not granted by the manufacturer. Although this affects only a single application, the findings may not fully represent all permanently listed DiGAs available at the time of the review. Furthermore, DiGAs are continuously updated after market entry. Consequently, the evaluated versions reflect the applications available during the review period, and subsequent updates may have altered functionalities, therapeutic content or user experience. The present review therefore represents a cross-sectional assessment of the included DiGAs rather than a permanent evaluation of their characteristics.

Interrater reliability for the guideline conformity assessment was substantial. This may partially be attributable to the structure of the applied assessment instrument, which allowed only the response options “yes”, “no”, or “to be determined”. Such limited response categories may reduce differentiation between partially and fully implemented criteria and increase interpretative variability between raters. The low agreement rate observed for the iCBT assessment should also be considered when interpreting these findings. Although all discrepancies were resolved through consensus discussions, future evaluations using this framework may benefit from more detailed rating guidance and standardized reviewer training to further improve interrater agreement.

Furthermore, the study focused exclusively on selected DiGAs for depressive disorders and did not evaluate long-term adherence or patient experiences in real-world settings. Therefore, conclusions regarding user engagement in routine care cannot be drawn from the present findings alone.

### 4.2 Implications for clinical practice

The variability identified across DiGAs treating depressive disorders highlights the need for more structured evaluation and comparison approaches within DiGAs. Given the increasing integration of DiGAs into clinical practice, physicians and psychotherapists require accessible, structured and transparent information regarding therapeutic content, functionality, monitoring approaches, and expected levels of user engagement in order to make informed prescription decisions.

Regardless of the psychotherapeutic approach (e.g. analytical, behavioral, systemic) a sustainable therapeutic connection is key to the effectiveness of psychotherapy (Norcross and Lambert, 2011). It is crucial for the patient to feel seen and heard as a person which may be challenging within the limitations of a more or less standardized mobile application. Taking into account the different clinical manifestations of depression, there is no “one fits all” solution. Hence, for clinicians being able to choose from applications that differ in content, depth, interactivity, and thematic focus may facilitate a more individualized approach to online psychotherapy. Broader information on the DiGAs available for prescription would contribute to a better quality in application based treatment of depression.

The present findings suggest that regulatory approval alone may not sufficiently capture clinically relevant differences between applications targeting the same disorder. Structured evaluation frameworks incorporating therapeutic, functional, and usability-related characteristics may therefore support clinicians in selecting DiGAs that align more closely with individual patient needs, symptom severity, and treatment preferences. Such studies could improve comparability between applications and facilitate more evidence-informed integration of digital interventions into routine care. Future research should investigate how differences in intervention structure, user involvement, and monitoring intensity influence adherence, patient satisfaction and clinical outcomes in real-world care settings.

## 5 Conclusion

The evaluated DiGAs for depressive disorders demonstrated high variability regarding the degree of guideline conformity, implementation of iCBT components, app quality, functional design and monitoring approaches. While several applications provided comprehensive and structured interventions, considerable differences were observed in therapeutic depth, interactivity, and supportive features.

These findings highlight the heterogeneity of currently available DiGAs for depression within the same regulatory framework. Greater transparency and more standardized evaluation approaches are therefore essential to improve comparability between applications and support informed decision-making in routine mental health care.

## Declaration of competing interest

The authors declare that they have no known competing financial interests or personal relationships that could have appeared to influence the work reported in this paper.

## Data Availability

The data that support the findings of this study are available from the corresponding author upon reasonable request.

## Appendix A Supplementary data

Supplementary Material S1 General application information

Supplementary Material S2 Guideline conformity

Supplementary Material S3 MARS

Supplementary Material S4 Functional characteristics
