# Supplementary material for: Digital therapeutics for depression in Germany: A systematic app review
Source: Internet Interv. 2026 Aug 25;46:100991. doi: 10.1016/j.invent.2026.100991 (PMC13527481; doi:10.1016/j.invent.2026.100991)
Supplement: Supplementary file 1 [file mmc1.pdf]

Supplementary Material S1: General Application Information

Category	Subcategory	deprexis	edupression.com	Novego: Depressionen bewältigen	My7steps App	Selfapys Online-Kurs bei Depression
**1. General information**	App languages	German, English, French, Italian, Spanish, Portuguese, Greek, Swedish, Chinese	German, English	German	Arabic, German English, French Greek, Italian Persian, Portuguese, Russian, Spanish, Turkish, Ukrainian	German, English
	Date of release (DiGA-directory)	February 20, 2021	December 26, 2022	October 10, 2021	February 17, 2023	December 16, 2020
	Riskclassification (MDR)	Risk class I according to MDR	Risk class I according to MDR	Risk class I according to MDR	Risk class I according to MDR	Risk class I according to MDR
	Date of last update	-	Google Play: 12.01.2026 iOS: 13.01.2026	-	-	Google Play:14.11.2025 iOS: 20.11.2025
	Availability of a desktop application	Yes	Yes	Yes	Yes	Yes
	Availability of a mobile phone application	No	Yes	No	No	Yes
	Used version number	6.1.	1.	Softwareversion 210	2.0	1.18.
**2. Operating System**	App exclusively for the iOS operating system	No	No	No	No	No
	App exclusively for the Android operating system	No	No	No	No	No
	App for both operating systems available	Yes	Yes	Yes	Yes	Yes

**3. Developer Information**	Name of the developer	GAIA AG, Germany	SOFY GmbH, Austria	IVPNetworks GmbH, Germany	My7steps GmbH, Germany	Selfapy GmbH, Germany
**4. Acquisition costs**	Freeware	228.00 €	224.80 €	199.00 €	207.00 €	217.18 €
**5. Target user groups**	Patients	Yes	Yes	Yes	Yes	Yes
	Patients and physicians	No	No	No	No	No
	Indications	F32.0; F32.1; F32. 2; F33.0; F33.1; F33.2	F32.0; F32.1; F33.0; F33.1	F32.0; F32.1; F32.2; F33.0; F33.1; F33.2; F34.1	F32.0; F32.1; F33.0 ; F33.1	F32.0; F32.1; F33.0; F33.1;
	Contraindications	F20; F23; F25; F31; R45.8	F21; F22; F31; F32.3; F33.3	F00; F01; F02; F03; F04; F05; F06; F07; F09; F10.2; F11.2; F12.2; F13.2; F14.2; F15.2; F16.2; F17.2; F18.2; F19.2; F20; F21; F22; F23; F24; F25; F28; F29; F30; F31	F20; F22; F23; F24; F32.2	F10.2; F10.3; F11.2; F11.3; F12.2; F12.3; F13.2; F13.3; F14.2; F14.3; F15.2; F15.3; F16.2; F16.3; F17.2; F17.3; F18.2; F18.3; F19.2; F19.3; F20; F21; F22; F23; F24; F25; F28; F29; F31; F32.2; F32.3; F33.2; F33.3
	female	Yes	Yes	Yes	Yes	Yes
	male	Yes	Yes	Yes	Yes	Yes
	non-binary	Yes	Yes	Yes	Yes	Yes

	Age	Adult(s) (18-65 years old), Adult(s) (over 65 years old)	Adult(s) (18-65 years old)	Adult(s) (18-65 years old)	Adult(s) (18-65 years old)	Adult(s) (18-65 years old), Adult(s) (over 65 years old)
**6. Interfaces**	Availability of an interface/connectivity to an external sensor(s)/device	No	No	No	No	No
**7. Data storage**	Whom belongs data?	Personal and health data belong to the data subject (user). GAIA AG is only responsible for processing in accordance with GDPR/HIPAA and uses the data exclusively for specific purposes. After the end of program use, all personal data is automatically and immediately deleted. In addition, users can request immediate deletion at any time; after deletion, access or export is no longer possible.	Although not explicitly stated in the documents, according to the GDPR, personal data remains the property of the data subject. The manufacturer processes the data as the controller only on the basis of contractual obligations and legal provisions.	Personal and health data belong to the data subject (user). The application manufacturer processes this data exclusively within the framework of legal provisions (in particular the GDPR and BDSG) and, where applicable, on the basis of contractual obligations.	Personal and health data belong to the data subject (user). The application manufacturer processes this data exclusively within the framework of legal provisions (in particular the GDPR and BDSG) and, where applicable, on the basis of contractual obligations.	Personal and health data belong to the data subject (user). The application manufacturer processes this data exclusively within the framework of legal provisions (in particular the GDPR and BDSG) and, where applicable, on the basis of contractual obligations.
	Where is data stored?	Exclusively in ISO 27001-certified data centers in Germany.	Storage location not specified; GDPR-compliant storage in secure systems.	The Novego application runs in a high-security center in Berlin.	Data is stored exclusively on servers within Germany or the	Personal and health data are processed and stored exclusively

					European Union. External service providers are only used in compliance with the GDPR and on the basis of corresponding data processing agreements. The exact location is not specified.	on secure severs of Deutsche Telekom. The data centers meet particularly high security standards and are located in Germany.
	How long is it stored? (general data affiliations)	General: as long as necessary for the contract/purpose, then deletion; for DiGA regulations, max. 90 days of use, then automatic and immediate deletion.	General: as long as necessary for the contract/purpose, then deletion. DiGA: automatic deletion of all personal data after 3 months, extension only with express consent.	Personal data will only be stored for as long as is necessary to fulfill the respective purposes or as required by statutory retention obligations. The specified storage periods refer explicitly to Novego participants. If the program booking is not completed, the prospective customer data will be completely	Personal data will only be stored for as long as is necessary to fulfill the respective purposes or as required by statutory retention obligations. No explicit deadline for automatic deletion is specified.	Personal data will only be stored for as long as is necessary to fulfill the respective purposes or as required by statutory retention obligations. No explicit deadline for automatic deletion is specified. With consent, data are stored until the consent is withdrawn. For contractual purposes, data are

				deleted after 6 months at the latest. When used as a “prescription app” (DiGA): storage for 365 days after redemption of the code; subsequent deletion after three months of inactivity. When used via health insurance, care program, or as a self-payer: storage for 12 months after completion of the respective program; subsequent deletion. Invoice data: Retention obligation 10 years in accordance with legal requirements (§147 (3) AO in conjunction with (1) No. 1, 4, and 4a AO, §14b (1) UStG); deletion after expiry of the deadlines.		retained for the duration of the contractual relationship.

**8. Security**	Password	Yes	Yes	Yes	Yes	Yes
	Two-factor authentication	No: No typical 2FA; authorization with Health ID possible	No: No typical 2FA; authorization with Health ID possible	No	No	No
	Additional security	TLS encryption for all data traffic; session timeout; hourly backups & disaster recovery tests; firewall and anomaly monitoring; regular external penetration tests; access only in a secure server environment; no storage of authentication data on end devices.	TLS-encrypted data transmission (applicable in the checklist); no data loss in the event of a power or Internet failure; plausibility check and consistency check of all entered or imported data.	TLS encryption; data transmission is carried out exclusively via SSL encryption; regular penetration tests; operation in a high-security center in Berlin (additional physical and organizational protective measures).	TLS-encrypted; TOM implemented according to the current state of the art; failure of power supply or Internet connection does not lead to data loss; plausibility checks are performed on entered data.	TLS-encrypted; TOM implemented; data transmission is carried out via SSL encryption.
