# Supplementary material for: Digital therapeutics for depression in Germany: A systematic app review
Source: Internet Interv. 2026 Aug 25;46:100991. doi: 10.1016/j.invent.2026.100991 (PMC13527481; doi:10.1016/j.invent.2026.100991)
Supplement: Supplementary file 2 [file mmc2.pdf]

Component	Category	Item	Subcategory	Novego: Depressionen bewältigen	Deprexis	Selfapy Online-Kurs bei Depression	My7Steps App	edupression.com
1. Psychoeducation and training	1.1 Information about the disease	1.1.1	Symptoms	Yes	Yes	Yes	Yes	Yes
		1.1.2	Diagnosis	Yes	Yes	Yes	No	Yes
		1.1.3	Cause(s)	Yes	No	Yes	Yes	Yes
		1.1.4	Course	No	No	No	No	Yes
		1.1.5	Biological, psychological and psychosocial aspects	Yes	Yes	Yes	No	Yes
		1.1.6	Treatability and prognosis	Yes	No	No	No	Yes
		1.1.7	Communication of a bio-psycho-social disease model to relieve patients of feelings of guilt, failure and self-blame	Yes	No	Yes	No	No
	1.2 Information on self-management	1.2.1	Connection between thinking, behaviour and feelings	Yes	Yes	Yes	No	Yes
		1.2.2	Sleep hygiene	Yes	No	No	No	Yes
		1.2.3	Anxiety management	Yes	Yes	No	No	No
		1.2.4	Stress management	No	No	Yes	No	Yes
		1.2.5	Communication and conflict resolution skills	Yes	Yes	Yes	No	Yes
		1.2.6	Day and lifestyle structuring (e.g. developing enjoyable activities)	Yes	Yes	Yes	Yes	Yes
		1.2.7	Learning relaxation techniques	Yes	Yes	Yes	No	Yes
		1.2.8	Dealing with the illness in a work context	Yes	No	No	No	No
	1.3 Information about the treatment	1.3.1	Mode of action, expected effects, possible interactions and side effects of antidepressants, latency, need for maintenance therapy, discontinuation symptoms and rebound phenomena	No	No	No	No	Yes
		1.3.2	Psychotherapy methods, effectiveness, need for continuation after symptoms have subsided, possible positive and negative side effects of psychotherapy	Yes	No	No	No	Yes
		1.3.3	Supporting services (sports and exercise therapies, light therapy, sleep deprivation therapy, etc.)	Yes	Yes	No	No	Yes
		1.3.4	Structured/complex forms of care	No	No	No	No	Yes
		1.3.5	Options in the event of non-response to treatment	No	No	No	No	Yes
		1.3.6	Neurostimulatory procedures	No	No	No	No	Yes
		1.3.7	Psychosocial therapies	No	No	No	No	Yes
		1.3.8	Benefits and potential harm of self-help groups	No	No	No	No	Yes
		1.3.9	Benefits of involving relatives and their involvement in relatives' groups	Yes	No	No	No	No
	1.4 Information on rehabilitative services, benefits for participation and other services	1.4.1	Medical rehabilitation	No	No	No	No	No
		1.4.2	Benefits for participation in work and education, benefits for social participation	Yes	Yes	Yes	No	Yes
		1.4.3	Services to support children, young people and families (household help, educational assistance, etc.)	No	No	No	No	No
		1.4.4	Information on counselling options regarding these services	No	No	Yes	No	Yes
	1.5 Relapse prevention and crisis management	1.5.1	Recognizing relapses and crises	Yes	No	Yes	No	Yes
		1.5.2	Dealing with suicidal tendencies	Yes	Yes	Yes	Yes	Yes
2. Cognitive behavioural therapy (CBT)		2.0.1	Individual problem analysis and therapeutic interventions that correct the problem behavior of depressed patients and develop an improved problem-solving repertoire	Yes	Yes	Yes	Yes	No
		2.0.2	Cognitive approaches aimed at alleviating depressive symptoms by identifying and changing dysfunctional attitudes and thinking and behavioral schema goals	Yes	Yes	Yes	Yes	Yes
	2.1 Acceptance and Commitment Therapy (ACT)	2.1.1	Behavioural therapy techniques combined with mindfulness- and acceptance-based strategies and interventions to clarify values	Yes	Yes	Yes	No	Yes
	2.2 Mindfulness Based Cognitive Therapy (MBCT)	2.2.1	Cognitive behavioural therapy techniques with mindfulness exercises	Yes	Yes	Yes	No	Yes
	2.3 Cognitive Behavioural Analysis System for Psychotherapy (CBASP)	2.3.1	Combines cognitive, behavioural, interpersonal and psychodynamic strategies	Yes	Yes	No	No	Yes
3. Psychonalytically based procedures[4]		3.0.1	Patients reflect on their feelings within a regression-promoting setting, primarily through "free speech"	No	No	No	No	No
		3.0.2	During treatment, psychotherapists observe their own, patient-related inner experience to better understand the patient and empathize with their inner experience, feelings and fears, verbalize them and address feelings that are unacceptable to the patient.	No	No	No	No	No
		3.0.3	Depressive experiences as well as concrete conflict actualizations in the current life situation	Yes	No	No	No	Yes
		3.0.4	Inner conflicts should be made conscious and the patients' abilities to overcome them should be used and strengthened.	Yes	No	Yes	No	No
4. Systemic therapies[3]		4.0.1	Focus on the social context of mental disorders	Yes	Yes	Yes	No	No
		4.0.2	Members of the social system that is important to the patient are often directly or indirectly involved in the treatment	No	No	Yes	No	No
		4.0.3	Using the strengths and resources of patients and relatives to change unfavourable behaviours, interaction patterns and evaluations, to support helpful communication and relationship patterns and to jointly develop solutions to existing problems and conflicts	Yes	No	Yes	Yes	No
		4.0.4	Circular or solution- and resource-oriented questions, paradoxical interventions or methods that explore the relationship experience of patients and their relatives	Yes	No	No	No	No
5. Cross-procedural impact factors for depressive disorders		5.0.1	Establishment of a sustainable therapeutic "working alliance"	Yes	No	No	No	No
		5.0.2	Clarification of motivation, goals, methods and setting of the treatment	Yes	No	Yes	Yes	Yes
		5.0.3	Determination of the individual treatment framework (implementation agreements, commitment of practitioners, duration, frequency and financing), taking into account the partial independence of this framework from ICD diagnoses	Yes	Yes	No	No	No
		5.0.4	Recognizing suffering as a prerequisite for change	No	No	No	No	No
		5.0.5	Experiencing unfavourable behavioural patterns within the therapeutic relationship and facilitating corrective learning experiences	No	No	No	No	No
		5.0.6	Enabling a deeper emotional experience	Yes	No	No	Yes	No
		5.0.7	Promotion of self-regulation and insight into relationship mechanisms and internal conflict and coping structures (especially avoidance of action and emotions)	Yes	Yes	Yes	No	Yes
		5.0.8	Clarification of the partial functionality of the symptoms	Yes	Yes	Yes	No	No
		5.0.9	Recognition and activation of personal and social resources (e.g. possible helpful actors in the patient's environment)	yes	Yes	Yes	Yes	Yes
		5.0.10	Initiating problem-solving and experiencing competence to promote self-esteem, self-congruence and self-efficacy, integrating what has been "understood" and "learned" into everyday life	Yes	Yes	Yes	Yes	No
6. Monitoring		6.0.1	When using internet and mobile-based interventions, adherence and effectiveness should be regularly monitored.	No	Yes	Yes	No	Yes
7. Therapeutic support		7.0.1	The use of Internet and mobile-based interventions should be accompanied by therapeutic support.	Yes	No	Yes	Yes	Yes
8. Low-intensity interventions (NVL 4.2)	8.1 Bibliotherapy	8.1.1	Handing out self-help or self-management literature	Yes	Yes	Yes	No	Yes
	8.2 Conversation-based interventions using psychotherapeutic techniques, e.g:	8.2.1	Psychoeducational-supportive conversations: explaining the illness; teaching simple self-help practices, e.g. regarding sleep-wake rhythm, daily structuring and anxiety management	Yes	Yes	Yes	No	Yes
		8.2.2	Counselling: psychosocial discussion and intervention techniques	Yes	Yes	Yes	Yes	No
		8.2.3	Problem-solving approaches: Teaching strategies for solving problems	Yes	Yes	Yes	No	Yes
		8.2.4	Behavioural activation: Reduction of avoidance and protective behaviour, inactivity, anhedonia and social withdrawal	Yes	Yes	Yes	No	Yes

Novego: Depressionen bewältigen	Deprexis	Selfapy Online-Kurs bei Depression	My7Steps App	edupression.com
Multi-component intervention combining: Psychoeducation and Behavioural activation exercises	Structured, dialogue-based format	Primarily text-based content	Structured as a seven-step pathway	Strong focus on psychoeducation
Content formats: text, audio, and video	Users select from predefined response options	Includes selected videos and conversational elements	Users progress through predefined modules	Each session includes: Educational component, recommendation (e.g., image with reflective sentence), short video content in app feed
Organized into 12 weekly lessons	Interactive conversational framework	Conversational elements alternate between: predefined response options, free-text input opportunities	Requires detailed screening at initial setup	Educational content is the core component: 59 videos of varying length, some videos exceed 40 minutes
Strong emphasis on increasing daily activities (activation focus)	User input limited to fixed answer choices	Interactive features present, but overall structure remains standardized	Users complete: Written reflections, Structured questions, Behavioural change tasks over defined time periods	Covers a wide range of topics in depth
Encourages consultation with a general practitioner	No option for free-text responses	User input does not influence: content sequence, therapeutic material	At predefined points, users are encouraged to consult assigned in-app therapists	Includes detailed explanations of pharmacological treatments and neurobiological mechanisms
Explicit recommendation to consider face-to-face psychotherapy after completion	Includes additional components beyond guideline criteria (e.g., content on dreams)	Despite multiple media formats, text remains dominant	Progression depends on sustained user engagement throughout the intervention	Includes: Neuroplasticity exercises, structured symptom queries
Includes additional topics beyond guidelines (Light therapy, wake therapy)	Some scenarios (e.g., party-related social situations) appear oriented toward younger users	Most exercises and reflective tasks available as downloadable PDFs		No continuous digital dialogue format
Covers a broad range of themes, including sensitive topics (e.g., sexual abuse)	Perceived relevance may vary depending on age and individual context of depression	Weekly PDF summaries provided		Exercises presented in an explanatory manner
Provides extensive downloadable supplementary materials				Limited step-by-step in-app guidance during exercises
				Emphasis on self-directed use of educational materials
				Provides downloadable materials: PDF summaries for 20 chapters, additional brochures for further reading
