# Supplementary material for: Digital therapeutics for depression in Germany: A systematic app review
Source: Internet Interv. 2026 Aug 25;46:100991. doi: 10.1016/j.invent.2026.100991 (PMC13527481; doi:10.1016/j.invent.2026.100991)
Supplement: Supplementary file 3 [file mmc3.pdf]

Mobile Application Rating Scale (MARS)

App Classification

App Name	Rating this version	Rating all versions	Developer	N ratings this version	N ratings all versions	Version	Last update	Cost - basic version	Cost - upgrade version	Plattform	Brief description	Focus	Affiliations	Age group	Technical aspects of app
deprexis	n/a	n/a	n/a	n/a	n/a	n/a	n/a	n/a	n/a	n/a	n/a	Depression	Commercial	Young Adults, Adults	Allows password-protection, requires login, sends reminders, needs web access to function
Selfapy - Online Kurs bei Depression	2,9	n/a	Selfapy GmbH	142	n/a	1.18.5	14.11.2025	n/a	n/a	Google Play	Selfapy is a digital app designed for people living with mental health conditions, offering methods and techniques based on cognitive behavioral therapy. The app helps you complete and track your exercises and provides helpful information on mental health and well-being.	Depression	Commercial	Young Adults, Adults	Allows password-protection, requires login, sends reminders, needs web access to function
edupression.com®	3,4	n/a	SOFY GmbH	60	n/a	1.10.23	04.03.2026	n/a	n/a	Google Play	edupression.com is a digital self-help therapy program for patients with major depressive disorder or burnout. The therapy is based on elements of behavioral therapy, as well as the latest scientific findings and methods.	Depression	Commercial	Young Adults, Adults	Allows password-protection, requires login, sends reminders, needs web access to function
My7stepps App	n/a	n/a	n/a	n/a	n/a	n/a	n/a	n/a	n/a	n/a	n/a	Depression	Commercial	Young Adults, Adults	Allows password-protection, requires login, sends reminders, needs web access to function
Novego: Depressionen bewältigen	n/a	n/a	n/a	n/a	n/a	n/a	n/a	n/a	n/a	n/a	n/a	Depression	Commercial	Young Adults, Adults	Allows password-protection, requires login, sends reminders, needs web access to function

Section A

Engagement

App	Entertainment	Interest	Customisation	Interactivity	Target group	Engagement mean score
deprexis	3,3	4,0	1,7	3,0	3,7	3,1
Selfapy - Online Kurs bei Depression	3,7	3,7	2,3	3,0	4,3	3,4
edupression.com®	3,0	3,33	2,00	2,67	2,00	2,6
My7stepps App	2,7	3,3	3,0	3,3	4,0	3,3
Novego: Depressionen bewältigen	3,7	4,0	2,7	3,0	4,0	3,5

Section B

Functionality

App	Performance	Ease of use	Navigation	Gestrural design	Functionality mean score
deprexis	5,0	4,7	4,3	5,0	4,8
Selfapy - Online Kurs bei Depression	4,0	4,0	3,7	4,0	3,9
edupression.com®	3,3	3,3	3,7	4,0	3,6
My7stepps App	4,0	4,3	4,3	4,7	4,3
Novego: Depressionen bewältigen	5,0	4,7	4,0	4,7	4,6

Section C

Aesthetics - graphic design, overall visual appeal, colour scheme, and stylistic consistency

App	Layout	Graphics	Visual appeal	Aesthetics mean score
deprexis	5,0	4,3	3,7	4,3
Selfapy - Online Kurs bei Depression	4,7	4,0	3,7	4,1
edupression.com®	3,67	3,33	3,33	3,4
My7stepps App	4,3	3,7	3,3	3,8
Novego: Depressionen bewältigen	4,7	4,0	3,3	4,0

Section D

Information - Contains high quality information (e.g. text, feedback, measures, references) from a credible source

App	Accuracy of app description (in app store)	Goals	Quality of information	quantity of information	visual information	Credibility	Evidence base	Information mean score
deprexis	5,0	4,3	4,3	4,3	4,7	4,0	5,0	4,5
Selfapy - Online Kurs bei Depression	4,0	3,7	4,7	4,7	4,3	4,0	4,3	4,2
edupression.com®	4,00	3,00	3,00	1,33	4,33	3,67	4,00	3,3
My7stepps App	5,0	3,7	3,3	3,0	4,0	4,0	3,0	3,7
Novego: Depressionen bewältigen	4,7	4,3	3,0	4,7	4,7	4,0	4,3	4,2

Section E

App subjective quality

App	Recommendation	Usage time in the next 12 months	Pay for this app	Overall star rating
deprexis	3,3	3,7	1,7	3,3
Selfapy - Online Kurs bei Depressior	3,3	3,0	2,3	3,0
edupression.com®	2,33	2,00	1,33	2,00
My7stepps App	2,7	2,3	1,3	2,7
Novego: Depressionen bewältigen	3,3	3,0	1,7	3,0

Scoring

App	Engagement Mean Score	Functionality Mean Score	Aesthetics Mean Score	Information Mean Score	App Quality Mean Score	App Subjective Quality Score
deprexis	3,1	4,8	4,3	4,5	4,2	3,0
Selfapy - Online Kurs bei Depression	3,4	3,9	4,1	4,2	3,9	2,9
edupression.com®	2,6	3,6	3,4	3,3	3,2	1,9
My7stepps App	3,3	4,3	3,8	3,7	3,8	2,3
Novego: Depressionen bewältigen	3,5	4,6	4,0	4,2	4,1	2,8

Section F (App-specific)

App	Awareness	Knowledge	Attitudes	Intention to change	Help seeking	Behaviour change
deprexis	4,0	4,0	3,3	4,3	2,3	3,7
Selfapy - Online Kurs bei Depression	4,3	4,0	4,0	4,0	3,3	3,7
edupression.com®	3,00	4,67	2,67	2,00	3,00	1,67
My7stepps App	2,7	2,3	3,3	2,7	3,3	2,7
Novego: Depressionen bewältigen	3,3	3,0	3,7	3,0	3,7	3,3
