# Supplementary material for: Digital therapeutics for depression in Germany: A systematic app review
Source: Internet Interv. 2026 Aug 25;46:100991. doi: 10.1016/j.invent.2026.100991 (PMC13527481; doi:10.1016/j.invent.2026.100991)
Supplement: Supplementary file 4 [file mmc4.pdf]

Supplementary Material S4: Functional characteristics

	deprexis	Novego: Depressionen bewältigen	My7steps App	Selfapys Online-Kurs bei Depression	edupression.com
Inform	1	1	1	1	1
Instruct	1	1	1	1	1
Record	1	1	1	1	1
Collect data	1	1	1	1	1
Share data	0	1	1	1	1
Evaluate data	1	0	1	0	1
Intervene	0	0	1	1	1
Display	1	1	1	0	1
Guide	0	1	1	0	1
Remind or alert	1	1	1	0	1
Communicate	0	1	1	1	1
Intervene	0	0	1	1	1
Display	1	1	1	0	1
Guide	0	1	1	0	1
Remind or alert	1	1	1	0	1
